# The roles and functions of Paneth cells in Crohn’s disease: A critical review

**DOI:** 10.1111/cpr.12958

**Published:** 2020-11-11

**Authors:** Erpeng Yang, Jun Shen

**Affiliations:** ^1^ Division of Gastroenterology and Hepatology Key Laboratory of Gastroenterology and Hepatology Ministry of Health, Inflammatory Bowel Disease Research Center Renji Hospital School of Medicine Shanghai Institute of Digestive Disease Shanghai Jiao Tong University Shanghai China

**Keywords:** Autophagy, Crohn's disease, Paneth cell, α‐defensins

## Abstract

Paneth cells (PCs) are located at the base of small intestinal crypts and secrete the α‐defensins, human α‐defensin 5 (HD‐5) and human α‐defensin 6 (HD‐6) in response to bacterial, cholinergic and other stimuli. The α‐defensins are broad‐spectrum microbicides that play critical roles in controlling gut microbiota and maintaining intestinal homeostasis. Inflammatory bowel disease, including ulcerative colitis and Crohn's disease (CD), is a complicated autoimmune disorder. The pathogenesis of CD involves genetic factors, environmental factors and microflora. Surprisingly, with regard to genetic factors, many susceptible genes and pathogenic pathways of CD, including nucleotide‐binding oligomerization domain 2 (NOD2), autophagy‐related 16‐like 1 (ATG16L1), immunity‐related guanosine triphosphatase family M (IRGM), wingless‐related integration site (Wnt), leucine‐rich repeat kinase 2 (LRRK2), histone deacetylases (HDACs), caspase‐8 (Casp8) and X‐box‐binding protein‐1 (XBP1), are relevant to PCs. As the underlying mechanisms are being unravelled, PCs are identified as the central element of CD pathogenesis, integrating factors among microbiota, intestinal epithelial barrier dysfunction and the immune system. In the present review, we demonstrate how these genes and pathways regulate CD pathogenesis via their action on PCs and what treatment modalities can be applied to deal with these PC‐mediated pathogenic processes.

## Graphical abstract

Paneth cells prevent Crohn's disease by secreting α‐defensins and other antimicrobial peptides. Genetic and environmental factors contribute to Paneth cell dysfunction and trigger Crohn's disease. Potential therapies against those risk factors can be used to treat Crohn's disease.

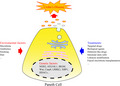

## 1 INTRODUCTION

Crohn's disease (CD) is a chronic inflammatory disease involving the gastrointestinal tract with symptoms such as abdominal pain, chronic diarrhoea, weight loss and fatigue typically.^1^ Although CD symptoms manifest in a relapsing and remitting manner, it is still a progressive disease, leading to bowel damage and disability.^1^ Although the cause and pathophysiology of CD remain unclear, it is believed to result from the interaction among genetic susceptibility, environmental factors and intestinal microflora, leading to an abnormal mucosal immune response and defective epithelial barrier.^1^

Many autophagy‐related genes, including NOD2, ATG16L1, IRGM, LRRK2 and XBP1, which also exert various effects on Paneth cells, were reported to be involved in IBD pathogenesis. Autophagy implies any cellular degradative pathway that delivers cytoplasmic cargo to lysosomes.^2^ Through autophagy, damaged organelles as well as invading bacteria can be engulfed by autophagosomes and sent to lysosomes for degradation, which is necessary for the activation of innate immunity.^3^ In addition to non‐selective degradation, autophagy can also selectively degrade specific targets. For example, NOD2 can specifically identify anti‐microbial peptides (AMPs) in PC vesicles and release them into the intestinal lumen instead of degradation.

The autophagy‐related genes play a variety of roles in CD pathogenesis. NOD2 is a pattern‐recognition receptor that maintains intestinal homeostasis.^3^ NOD2 functions through mechanisms including autophagy, intracellular bacterial sensing, improving immune tolerance by suppressing Toll‐like receptor (TLR) signals, regulating the expression of α‐defensins in PCs and recruiting ATG16L1 to the plasma membrane at bacterial entry sites.^4^ ATG16L1 is a homolog of ATG16 that is involved in the expansion and closure of the autophagosome membrane.^5^ IRGM participates in inflammation regulation and autophagy activation and is associated with bactericidal effect, vacuolar trafficking and acidification, phagosome maturation and virus‐induced autophagy.^2^ LRRK2 is a well‐ known Parkinson's disease (PD) susceptible locus. LRRK2 deficiency deregulates autophagy, leading to PC defects.^6^ Finally, XBP1, a transcription factor, is an unfolded protein response (UPR) inducer that regulates PC level.^7^

Additionally, other genes also participate in regulating PCs and preventing CD. First, Wnt signalling mediates PC differentiation and its associated transcription factors, including T‐cell factor 1 (TCF‐1), T‐cell factor 4 (TCF‐4) and lipoprotein receptor‐related proteins 6 (LRP6), and also regulates α‐defensin expression in PCs.^8^, ^9^ Second, HDACs influence PC phenotype and are related to the loss of PC functions.^10^, ^11^ Finally, Casp8, a well‐known apoptosis‐mediated protein, also found to be associated with PC death in CD.^12^

In the present review, we sequentially demonstrate how each of these genes and pathways interacts with environmental factors and regulates CD pathogenesis via their effects on Paneth cells. Subsequently, potential therapeutic targets dealing with these PC‐mediated pathogenic processes will be discussed.

## 2 NOD2 PREVENTS CD THROUGH DIRECTLY REGULATING α‐DEFENSINS EXPRESSION AND AMP SORTING IN PCs

**Figure 1:**
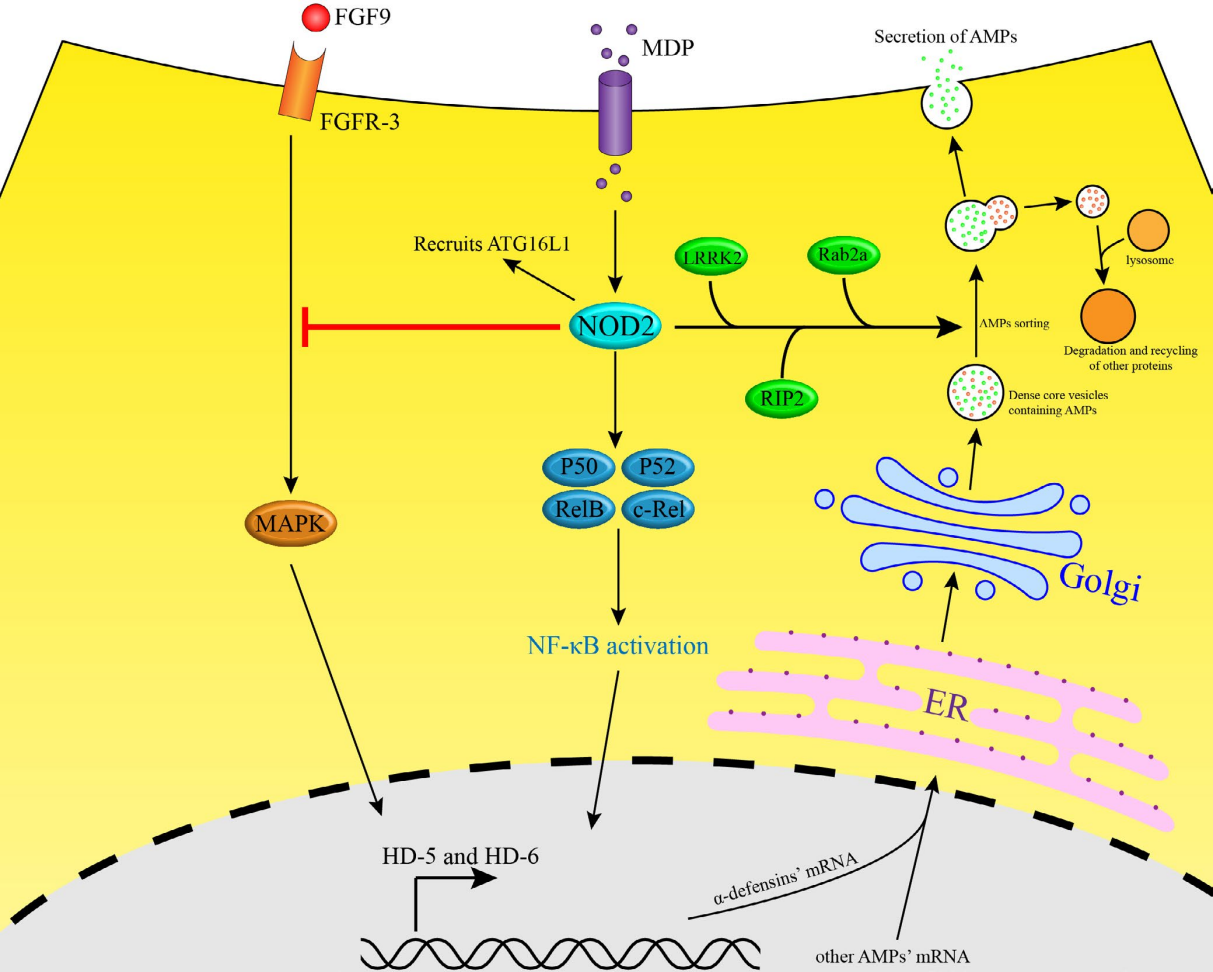
NOD2 regulates AMP α‐defensins expression and AMPs sorting in PCs. First, NOD2 directly regulates α‐defensin expression through the NF‐κB pathway. Furthermore, FGFR‐3 mediates α‐defensin expression through the MAPK pathway which is inhibited by NOD2. In addition, NOD2‐LRRK2–RIP2‐RAB2a pathway is responsible for AMP sorting. At last, NOD2 recruits ATG16L1 to initiate autophagy

Nucleotide‐binding oligomerization domain 2 (NOD2) is a 110‐kDa cytosolic protein with two card domains and belongs to the NOD‐like receptor (NLR) family.^13^, ^14^ Genome‐wide association studies (GWAS) have identified NOD2 as a major risk factor for ileal CD.^15^, ^16^ Furthermore, according to a meta‐analysis, the risk of developing CD is increased to 17.1‐fold in individuals with two mutated NOD2 alleles and 2.4‐fold in simple NOD2 heterozygotes.^17^ As a protective factor of CD, NOD2 exerts a variety of functions and is expressed in many kinds of immune cells. Notably, NOD2 is highly expressed in ileac PCs, associated with α‐defensin expression.^18^, ^19^ In addition, several prior studies showed that Paneth cell is a central location in which NOD2 functions to prevent CD. The mechanisms involved may include the regulation of nuclear factor κB (NF‐κB) and mitogen‐activated protein kinase (MAPK) pathways, lysozyme sorting and the recruitment of ATG16L1.^14^, ^20^ Two major functions of NOD2 in PCs will be introduced (Figure 1).

First, NOD2 regulates the expression of human enteric α‐defensins (HD‐5 and HD‐6) in PCs.^21^ The proper expression of α‐defensins is crucial in preventing CD. Prior studies have shown that NOD2 plays varying roles in upregulating or downregulating α‐defensin on different conditions.^21^, ^22^ NOD2 activation can slightly upregulate α‐defensin expression through the NF‐κB pathway.^21^ In the experiments conducted in a prior study, Caco2 intestinal epithelial cells were used instead of human PCs because the latter do not survive in vitro.^19^, ^23^ Also, muramyl dipeptide (MDP), an agonist of NOD2,^24^ was used to activate NOD2. The results obtained showed that only MDP‐stimulated Caco2 cells with wild‐type NOD2 expressed a significantly higher level of HD‐5 and HD‐6. Furthermore, when MDP‐stimulated Caco2 cells were treated with NF‐κB inhibitor BAY117082, the upregulation of α‐defensins was blocked.^21^ As anticipated, some of the NF‐κB subunits (p52, p50, RelB and c‐Rel) that were isolated from MDP‐stimulated Caco2 cells showed significantly increased DNA‐binding activity.^21^ Hence, it was suggested that NOD2 itself upregulates α‐defensin expression through the NF‐κB pathway. However, during the differentiation of the PC lineage, AMP expression may be downregulated by NOD2.^22^ During PC differentiation, fibroblast growth factor receptor 3 (FGFR‐3) is highly expressed and plays a critical regulatory role.^25^, ^26^ FGF‐9, a high‐affinity ligand of FGFR‐3, was used to trigger FGFR‐3‐mediated signalling in Caco2 cells along PC lineage.^22^ The data showed that activated FGFR‐3 mediates α‐defensin expression via the MAPK pathway without NOD2 activation.^21^ Nevertheless, the additional application of MDP significantly decreased AMP expression including HD5, HD6, lysozyme and sPLA2.^21^

Second, NOD2 participates in PC‐derived AMP sorting. After AMPs are synthesized in the ER, they need to be sorted into specialized dense core vesicles (DCVs) in Golgi network.^27^ After DCVs budding off from the Golgi network, AMPs will be retained in DCVs while non–DCV‐destined cargos are picked out and directed for degradation.^20^ Although the detailed mechanisms of sorting are unknown, experimental data suggested that the NOD2‐LRRK2–RIP2‐RAB2a pathway may play a critical role in it.^20^ At the outset, intestinal bacteria are necessary for the initiation of cargo sorting in PCs.^28^ Next, NOD2 recruits LRRK2 to the DCV surface. Next, commensal bacterium–derived signalling triggers NOD2 to complex with RIP2 while LRRK2 enhances and stabilizes formation of the complex.^20^, ^29^ Finally, Rab2a may be recruited directly or indirectly to the DCV surface by RIP2.^20^ In summary, AMPs will be directed to lysosomes instead of staying in DCVs if NOD2 is deficient. Thus, CD may be triggered by a lack of PC‐derived AMPs in NOD2‐deficient patients.

## 3 ATG16L1 EXERTS PROTECTIVE EFFECTS IN CD BY MAINTAINING AUTOPHAGY AND RESPONDING TO ENVIRONMENTAL STRESS IN PCs

**Figure 2:**
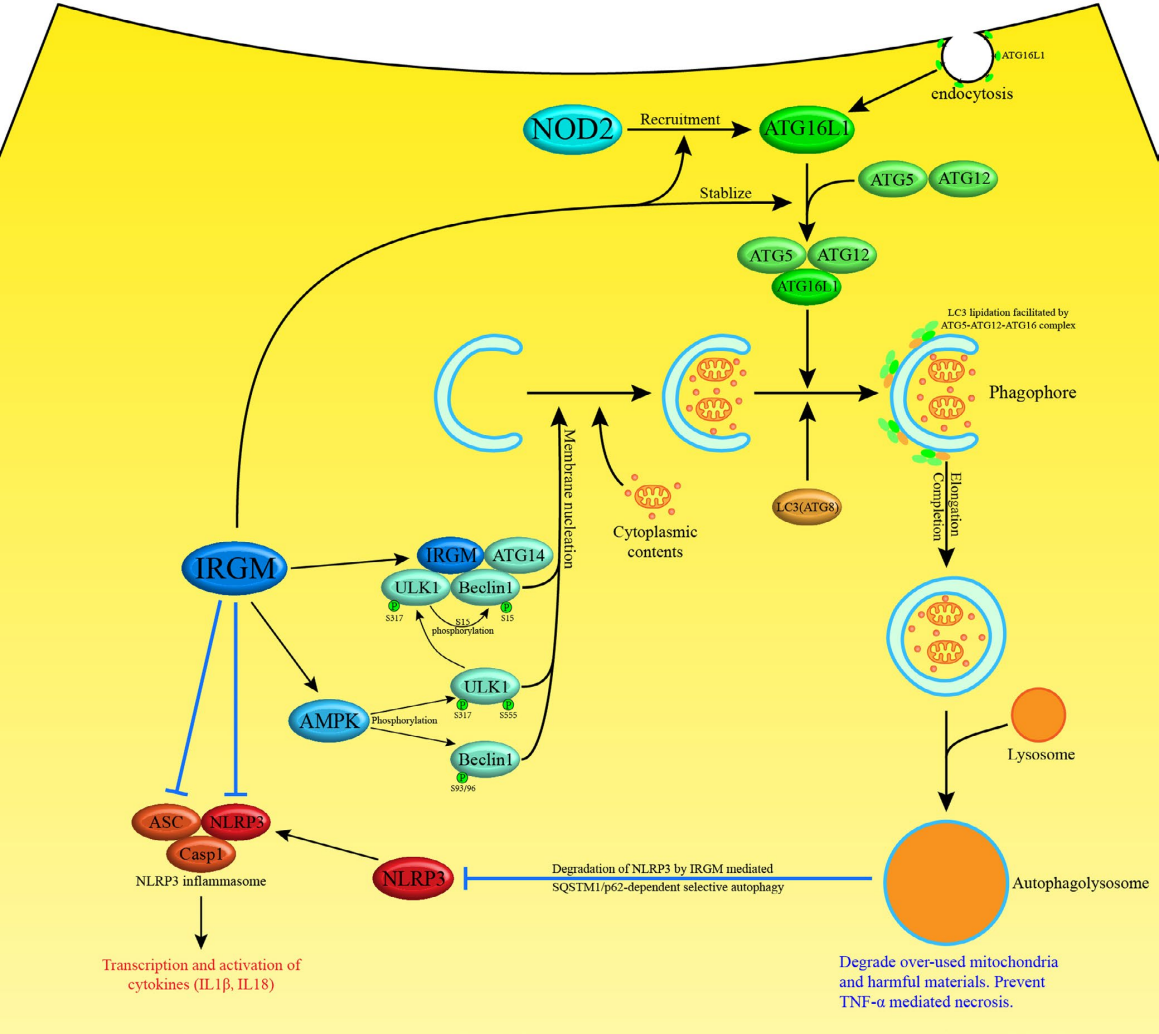
ATG16L1 and IRGM prevent CD through regulating autophagy and inhibiting inflammation. To begin with, ATG16L1 regulates autophagosome expansion and closure through mediating LC3 lipidation with the aid of ATG5 and ATG12. Furthermore, IRGM initiates the phosphorylation cascade that activates ULK1 and Beclin 1, which promotes autophagy. Finally, IRGM inhibits the NLRP3 inflammasome through mediating SQSTM1/p62‐dependent selective autophagy of NLRP3 and obstructing the polymerization of NLRP3 and ASC

To date, multiple protective effects of ATG16L1 in IBD have been discovered, including enhancing Th1/Th17 response,^30^ facilitating host‐bacteria interactions in myeloid cells^31^ and particularly preventing PC abnormality and death. The key role ATG16L1 played in autophagy in PCs was firstly reported in 2008. According to this study, ATG16L1‐deficient PCs of mice exhibited striking abnormalities in the granule exocytosis pathway while the PCs of CD patients with homozygous ATG16L1 risk allele showed similar abnormalities (expressing an increased level of leptin protein).^32^ Subsequently, the exact functions of ATG16L1 in PC autophagy were gradually elucidated (Figure 2). ATG16L1 participates in the formation of autophagosome. Formation of autophagosome is enabled by approximately 30 proteins and microtubule‐associated protein light chain 3 (LC3), an autophagy‐related protein 8 (ATG8) family protein, is a key player among them.^33^ Similar to most proteins, LC3 needs to be activated to function normally. After the C‐terminal of the LC3 precursor is cleaved, lipid phosphatidylethanolamine needs to conjugate with the glycine residue exposed on the new C‐terminal.^34^, ^35^, ^36^ This process is termed LC3 lipidation which appears to mediate the expansion and closure of the autophagosome membrane. Like ubiquitin, the reaction is facilitated by ATG7 (E1‐like enzyme), ATG3 (E2‐like enzyme) and the ATG5‐ATG12‐ATG16 complex (E3‐like enzyme).^37^, ^38^

Based on a recent study, autophagy impairment resulting from ATG16L1 deficiency alters the proteomic abundance profiles in PCs.^39^ In the experiment conducted, the levels of 283 proteins (corresponding to 284 human orthologue proteins) were detected in PC‐rich organoids derived from both WT and ATG16L1 deficiency mice. The results obtained showed that the abundance of 116 proteins was significantly altered, 81 of which were ATG16L1 targeted, indicating that a variety of proteins and functions can be influenced by ATG16L1.^36^ More importantly, after proteins with altered abundance were classified based on 16 major cell functional processes, it was identified that exocytosis was inhibited.^39^ As is mentioned above, defensins and antibacterial peptides were largely secreted by PCs through exocytosis. Hence, the malfunction of ATG16L1‐deficient PCs may trigger CD.

Furthermore, ATG16L1 prevents TNF‐α‐mediated PC necroptosis by maintaining mitochondrial homeostasis.^5^ As is widely reported, ATG16L1 deficiency results in autophagy impairment. One of the functions of autophagy is to recycle mitochondria when reactive oxygen species (ROS) accumulates.^40^ According to one particular study, mitochondria that accumulated a significant amount of ROS and failed to degrade through autophagy contributed to the susceptibility of PCs to TNF‐α‐mediated necroptosis.^5^ As was anticipated, necrotic PC death and mitochondrial abnormality were found in ATG16L1^ΔIEC^ organoids.^5^ Surprisingly, rescue effects were found in both necrostatin‐1 (Nec‐1) and antioxidant *N*‐acetyl‐l‐cysteine (NAC).^5^ Nec‐1 is an inhibitor of RIPK1^41^ that interrupts the TNF‐α pathway and NAC can help clean ROS in mitochondria. Furthermore, the sole deletion of Parkin (Park2), a protein on the mitochondrial membrane that directs autophagy degradation,^42^ made organoids susceptible to TNF‐α‐induced death.^5^

Additionally, the ATG16L1 gene can interact with environmental factors to influence PC function. First, ATG16L1‐mediated autophagy may be upregulated by starvation.^34^ Before ATG16L1 is recruited to phagophores, ATG16L1 proteins that bind to the plasma membrane, a major source of ATG16L1,^43^ have to be internalized through endocytosis, and the fusion of ATG16L1‐containing vesicles to other vesicles (eg ATG9A‐containing vesicles) needs to be directed.^34^ When cells are exposed to starvation, JNK is activated, which enhances the binding of c‐Jun to its promoter.^44^ Following this, Annexin A2 transcription is upregulated, which may lead to increased endocytosis and homotypic fusion of ATG16L1‐containing vesicles.^45^ Second, smoking interacts with the ATG16L1^T300A^ gene to trigger PC defect in CD. ATG16L1^T300A^ is the most common CD‐susceptible SNP in Caucasian patients.^3^ According to a CD cohort study, smokers with ATG16L1^T300A^ allele have a significantly lower percentage of normal PCs compared with the percentage of PCs seen in other groups.^46^ Experiments conducted in mice models showed the same result,^46^ indicating a synergistic effect between ATG16L1^T300A^ gene and smoking. To confirm the underlying mechanism, a transcriptomics analysis was performed. The results obtained indicated that the proliferator‐activated receptor‐gamma (PPARγ) pathway is a central mechanism that results in PC defect in ATG16L1^T300A^‐smoking CD patients.^46^ PPARγ exerts a variety of functions, including regulation of metabolism, differentiation and cell growth.^47^ The transcription of PPARγ pathway genes was downregulated in ATG16L1^T300A^‐smoking mice and CD subjects, leading to crypt cell and PC apoptosis. These findings were bolstered by the rescue effect of the PPARγ agonist rosiglitazone on the apoptosis of ATG16L1^T300A^‐smoking mice.^46^ Finally, ATG16L1 can specifically interact with murine norovirus (MNV).^48^ According to a prior study, hypomorphic (HM) ATG16L1 mice infected by MNV CR6 showed morphological and granule‐packaging abnormalities.^48^ However, several factors, including TNF‐α antibody, IFN‐γ antibody and antibiotics, can rescue mice from intestinal injury, indicating that TNF‐α, IFN‐γ and commensal bacteria may play roles in virus‐susceptible gene interaction.^48^

## 4 IRGM GOVERNS THE CORE AUTOPHAGY MECHANISMS THAT MEDIATE ANTI‐INFLAMMATION AND ANTI‐MICROBIAL FUNCTIONS IN INTESTINAL EPITHELIAL CELLS, REDUCING THE RISK OF CD

Immunity‐related guanosine triphosphatase family M (IRGM), a protein that mediates inflammation and autophagy in PCs, was revealed to exert a protective effect in CD (Figure 2).

First, IRGM negatively regulates the transcription of pro‐inflammatory cytokines (IL‐1β, IL‐18, and TNF/TNF‐α) through inhibiting the NLRP3 inflammasome.^49^ A functional NLRP3 inflammasome is composed of NLRP3, ASC (apoptosis‐associated speck‐like protein containing a CARD) and caspase‐1. NLRP3 complexes with ASC to activate CASP1, which in turn cleaves and activates the precursors of pro‐inflammatory cytokines such as IL1β.^50^ IRGM utilizes two parallel independent approaches to limit the activity of NLRP3 inflammasomes.^49^ First, IRGM can directly bind to the oligomerization domains of NLRP3 and ASC to obstruct their polymerization, leading to compromised formation of productive inflammasomes. Second, IRGM can mediate SQSTM1/p62‐dependent selective autophagy of NLRP3 and PYCARD, leading to reduced inflammasome numbers in the cell.^49^

Immunity‐related guanosine triphosphatase family M can also play a direct role in organizing the core autophagy machinery to endow it with anti‐microbial and anti‐inflammatory functions.^51^ First, IRGM is capable of initiating the phosphorylation cascade that activates ULK1 and Beclin1, which promotes autophagy. During this process, IRGM complexes with ULK1 and Beclin1 and activates AMPK, which in turn activates ULK1 and Beclin1. Furthermore, Beclin1 can interact with other molecules to activate the initiator complex of autophagy.^52^ Second, IRGM, NOD2 and ATG16L1 form a molecular complex to modulate autophagic responses to microbial products.^51^ IRGM works as an adaptor that is of great importance in promoting the assembly of the complex.

Inflammation disorder can be alleviated by IRGM via the pathways described above, which is in accordance with the results of experiments on IRGM1 knockout mice.^53^ PC autophagic deficiency was detected in IRGM1 knockout mice. Intestinal inflammation also occurred after the exposure of mice to dextran sodium sulphate.^53^

However, according to a statistical study, IRGM failed to show relevance to CD in an Iranian population, while the association between ATG16L1 and CD was confirmed,^54^ indicating genetic diversity among populations.

## 5 WNT SIGNALLING ABNORMALITY ELIMINATES PC DEFENSIN PRODUCTION AND TRIGGERS CD

Wnt signalling plays an important role in regulating cell fate and differentiation.^55^ Among intestinal cells, Wnt signalling regulates the positioning, differentiation and maturation of PCs.^56^, ^57^ The Wnt signalling pathway can be triggered when Wnt family proteins bind to cell‐surface receptors, stabilizing cytoplasmatic β‐catenin.^56^ Following this, the stabilized β‐catenin translocated into the nucleus, forming a complex with transcription factors of the T‐cell factor/lymphoid enhancer‐binding factor (Tcf/Lef) family and activates various target genes.^56^ According to prior researches, elements of Wnt signalling pathway, including TCF‐1, TCF‐4 and LRP6, regulate α‐defensins expression in PCs and, therefore, are susceptible loci for CD.

TCF‐1, one of the downstream transcription factors in the Wnt pathway, directly regulates HD‐5 and HD‐6 expression in PCs.^8^ Three TCF consensus elements, functioning as TCF‐1 binding sites, in both HD‐5 and HD‐6 promoters are responsible for their activation.^8^, ^58^ Notably, among these TCF‐binding sites (‐113, ‐130, ‐159 in HD‐5 and ‐130, ‐141, ‐159 in HD‐6), position −130 plays a prominent role in the activation process. In contrast to other positions, −130 mutation significantly reduced HD‐5 expression and nearly completely abolished HD‐6 expression induced by TCF‐1.^8^ Furthermore, the level and activity of β‐catenin, a cofactor of TCF‐1, may also be a limiting factor of TCF‐1‐induced α‐defensin expression. Finally, in both adult and child CD patients, the expression of TCF‐1 and active TCF‐1 isoforms was both significantly reduced,^8^, ^59^ confirming its role in CD pathology.

Likewise, the decreased expression and reduced binding activity of TCF‐4 are linked to reduce α‐defensin, expression in the PCs of ileal CD patients independent of NOD2, IL‐8 and inflammation.^9^ Similar to TCF‐1, as was reported, TCF‐4 has five potential binding sites in the HD5 promoter region and 11 in the HD6 promoter region, allowing TCF‐4 to regulate their transcription directly.^56^, ^60^ As a result, mutations in TCF‐4 may be risk factors for CD due to differentiated PC secretion of α‐defensins. According to a high‐throughput analysis in three IBD cohorts from Oxford, Leuven and Vienna,^61^, ^62^, ^63^ an association between a TCF‐4 SNP and ileal CD was reported. A susceptible SNP in the putative promoter region of TCF‐4, rs3814570, is associated with decreased expression of TCF‐4 and ileal CD phenotypes.^64^ Patients with rs3814570 alleles showed higher risk of stricturing ileal CD and upper GIT involvement (L4 phenotype).^64^

Furthermore, a low‐density lipoprotein receptor‐related protein 6 (LPR6) non‐synonymous SNP (rs2302685; Ile1062Val) is associated with reduced HD‐5 expression in PCs and the early onset of ileal CD.^65^ LPR6 is a co‐receptor of Wnt and is fundamental for cytoplasmatic stabilization of β‐catenin.^66^ The functional impairment of LPR6 may diminish the effect of Wnt downstream transcription factors TCF‐1 and TCF‐4, resulting in a lower HD‐5 expression level.

## 6 LRRK2 DEFICIENCY DEREGULATES AUTOPHAGY IN PCs

Leucine‐rich repeat kinase 2 (LRRK2) is known as a PD susceptible loci. However, LRRK2 single nucleotide polymorphisms were also proposed as risk loci for CD.^67^ According to a prior study, rs17467164, rs11564258 and rs3761863 were revealed to be related to the CD phenotype.^67^ Although the mechanism behind how LRRK2 deficiency affects CD is still unknown,^68^ much evidence supports the fact that LRRK2 plays a role in mediating autophagy in PCs. First, LRRK2 is a shared susceptible loci for PD and CD. LRRK2 deficiency can result in the deregulation of autophagy in PD, indicating that the underlying mechanism may be the same in CD. Second, according to an in vivo experiment, LRRK2 deficiency mice showed a specific impairment in the expression of lysozyme in PCs.^28^ Finally, in a Japanese CD cohort, the hypothesis‐driven correlation analysis showed significant association between LRRK2 M2397T SNP (rs3761863) and PC defects.^6^ Also, correlation was found between numbers of the T (risk) allele *LRRK2* M2397T and the percentage of normal PCs (*R* ^2^ = 0.247; *P* = 3.62 × 10^‐4^).^6^

## 7 HDACs REGULATE THE INTRINSIC PC PHENOTYPE AND ARE RELATED TO PC FUNCTION LOSS IN CD

Histone deacetylase (HDAC) is an enzyme that regulates transcription, DNA replication and repair. There are more than 10 subtypes of HDAC altogether, among which HDAC1, HDAC2 and HDAC3 were reported to be related to PCs and CD.

Although HDAC1 and HDAC2 act through different regulatory pathways, HDAC1 and HDAC2 may complement each other to regulate the intrinsic PC phenotype.^10^ HDAC1 deletion in mice leads to embryonic lethality^69^ while HDAC2 deficiency in mice results in perinatal lethality stemming from heart defects.^70^ Additionally, in the intestine, IEC‐specific HDAC1 and HDAC2 villin‐Cre‐induced deletion results in PC loss, the activation of the Notch, Stat3 and mTOR pathways, as well as increased susceptibility to DSS‐induced colitis.^71^, ^72^ However, in vivo, it has been previously observed that IEC‐specific HDAC1‐ or HDAC2‐deficient mice do not display intestinal architectural defects,^10^ indicating the complementary role HDAC1 and HDAC2 play. Additionally, HDAC1 and HDAC2 deficiency mice displayed several symptoms, including PC differentiation alterations, reduction of secretory PCs in the jejunum and intestinal chronic inflammation.^71^ According to another study, when HDAC1 or HDAC2 was deleted, a significant augmentation in the number of intermediate cells (precursor of both goblet cells and PCs) displaying both goblet and PC labels was also observed, revealing that HDAC1 and HDAC2 can influence the differentiation of PCs.^10^ Although some of the mechanisms involved are unclear, a variety of pathways may influence the process. For example, HDAC1 and HDAC2 can regulate Notch, STAT1, STAT 3, mTOR and NF‐κB pathways.^10^, ^73^, ^74^ All these pathways play important roles in cellular activities.

In addition to HDAC1 and HDAC2, HDAC3 was also found to be related to PC function loss and CD. As was revealed, HDAC3 is a critical factor that integrates commensal bacteria‐derived signals to calibrate epithelial cell responses required to establish normal host‐commensal relationships and maintain intestinal homeostasis.^11^ In the present study, a significant decrease in numbers, reduced lysozyme expression, the presence of degenerating organelle membranes and loss of granules were observed in the PCs of HDAC3^ΔIEC^ mice.^11^ Consistent with the lack of PCs, HDAC3^ΔIEC^ mice also exhibited impaired crypt bactericidal activity and increased susceptibility to oral *Listeria monocytogenes* infection.^11^ Furthermore, in contrast to the germ‐free condition, in the conventionally housed condition, WT mice colonized with either the HDAC3FF or HDAC3^ΔIEC^ microbiota exhibit significant differences in susceptibility to DSS‐induced intestinal inflammation, indicating that HDAC3 expression is required to integrate signals derived from commensal bacteria.^11^

## 8 CASP‐8 RESTRICTS PANETH CELLS DEATH IN INTESTINAL INFLAMMATION

**Figure 3:**
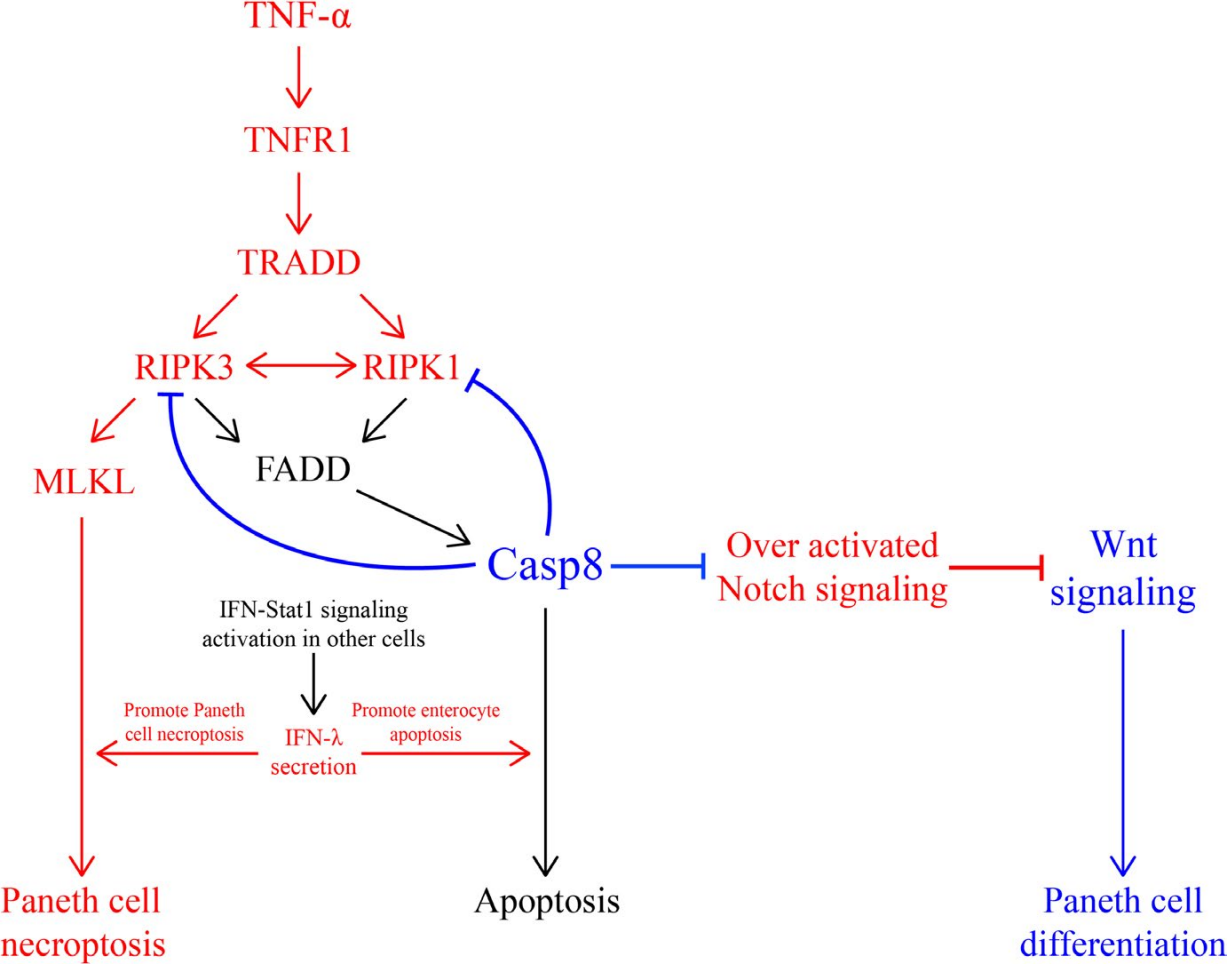
Casp8 plays a central role in preventing PC necroptosis. Casp8 inhibits TNF‐α induced necroptosis through suppressing RIPK1 and RIPK3 and enhances Wnt signalling by preventing Notch overactivation. Additionally, IFN‐STAT1 signalling may trigger enteric cell loss through activating MLKL and Casp8

Casp‐8 is a cysteine protease known for its critical role in regulating apoptosis. Additionally, various functions of Casp8 were elucidated, including proliferation, migration and differentiation.^75^, ^76^ In intestine, Casp8‐mediated apoptosis is important for the IEC renewal and for shaping the intestinal morphology.^77^ Also, PCs are interfered by Casp8 crucially in these processes (Figure 3).

First, Casp8 plays a critical role in preventing necroptosis of PCs and terminal ileitis by inhibiting TNF‐α‐induced necroptosis through suppressing RIPK1 and RIPK3.^12^ It is indicated that Casp8^ΔIEC^ mice suffered heavy Paneth and goblet cell loss. Additionally, on exposure to DSS, Casp8^ΔIEC^ mice showed high lethality and lost significantly more weight compared with the control mice.^12^ The mechanisms behind how Casp8 prevents PC loss can be explained by the inhibition of the TNF‐α pathway which induces cellular necroptosis through activating TRADD and RIPK3. After TNF‐α is upregulated, TRADD is activated by the receptor of TNF‐α (TNFR1).^78^ Then, receptor‐interacting protein kinase 3 (RIPK3), which is recruited to RIP1 to establish a necroptosis inducing protein complex, can be activated by TRADD.^78^ Finally, necroptosis can be stopped through inhibiting RIPK1 and RIPK3 by Casp8. TNF‐α stimulated death receptor signalling has been described to promote necrosis in a number of different target cell types, especially when apoptosis was blocked using caspase inhibitors.^79^, ^80^ According to some prior studies, when a dose of TNF‐α that is not lethal to normal mice was administered to Casp8^ΔIEC^ mice, high lethality and increased number of dying small intestinal epithelial cells (including PCs) were observed,^12^ indicating that TNF‐α can also promote PC death.

Secondly, PC death due to Casp8 deficiency is strongly associated with Notch activation.^81^ The Notch signalling pathway is a cell‐cell communication process that enhances intestinal homeostasis by participating in enterocytic proliferation, differentiation and apoptotic death.^82^ Currently, Notch signalling is viewed as an antagonistic pathway to Wnt/β‐catenin signalling while they reciprocally maintain intestinal homeostasis.^83^ As mentioned above, the differentiation of PCs can be promoted by the Wnt pathway. Recently, the role played by Notch in casp8^∆int^ animals in inhibiting secretory cell differentiation and diminishing PC number was elucidated.^81^ In a casp8^∆int^ mouse model, PC loss as well as strong Notch signalling activation was found. Nevertheless, when Notch was inhibited by the injection of a dose of 20 μM dibenzazepine (DBZ) per kg body weight, a dramatic expansion of secretory cells was observed, which indicates differentiation of PCs.^81^ Additionally, other proteins are also involved in Notch signalling in PCs. Based on another study, a tryptophan metabolizing enzyme, indoleamine 2, 3‐dioxygenase 1 (IDO1), promotes PC differentiation by inhibiting Notch 1 pathway activation.^84^ Furthermore, some proteins that regulate autophagy (eg ATG16L1) may also inhibit Notch by promoting its degradation.

Third, another non‐canonical PC necrosis pathway, the IFN‐STAT1 signalling, was proved to be related to Casp8 deficiency CD.^85^ IFN lambda (IFNL) is a multifunctional interferon that can be upregulated by other IFNs secreted by immune cells.^86^ In both type 1 and type 2 PCs, IFNL mRNA expression was elevated.^85^ According to a recently published study, it was observed that the expression levels of IFNL and STAT1 (target of IFNL) correlate well with CD severity and necrotic PC death.^85^ Surprisingly, a Stat1‐dependent role that Mixed Lineage Kinase Domain Like (MLKL) plays in PC programmed necrosis was discovered.^85^ After being phosphorylated by RIPK3,^87^ MLKL can translocate to the plasma membrane and mediate its destruction, which may induce necrosis in PCs.^88^ In Casp8^ΔIEC^ mice, MLKL acts as a complement for Casp8 that directs PC death.^85^ However, excessive necrosis of PCs that induced by MLKL may be a major cause for intestinal inflammation. Thus, the critical role Casp8 plays in maintaining intestinal homeostasis and regulating PC death has been identified.^89^

## 9 XBP1 IS NECESSARY FOR PC DEVELOPMENT

X‐box‐binding protein‐1 (XBP1), a transcription factor, is an UPR inducer that is processed by IRE1.^90^ After the excision of a 26‐bp mRNA fragment of XBP1 directed by IRE1, XBP1s is unconventionally produced.^90^ A subset of UPR genes expression can be induced by XBP1s.^90^ XBP1 Activation is necessary for ER expansion,^91^ which is crucial for the development of secretory cells^92^ such as PCs. An IEC XBP1^−/−^ mouse model showed that both PC loss and spontaneous IBDs (including CD) can be triggered by XBP1 deficiency. XBP1 regulates PC level through preventing apoptosis and mediating cell renewal,^7^ while the disruption of which directly leads to impaired mucosal defence. Inflammation in IECs that may contribute to PC apoptosis is triggered by both infection and upregulation of typical IEC inflammatory signals.^7^ To be specific, XBP1 deficiency in the small intestinal epithelium enhances pro‐inflammatory JNK/SAPK signalling in IECs.^7^, ^93^

Many SNPs in the XBP1 gene region are associated with CD. After analysing data from several cohorts containing 5322 controls, 2762 CD and 1627 UC patients,^7^, ^94^ a total of 19 SNPs were found to be significantly related to both CD and UC. Among these, six SNPs (rs5997391, rs5752792, rs6005863, rs5762795, rs2267131 and rs35873774) showed significantly stronger relevance.^7^

## 10 THERAPEUTIC MECHANISM TARGETING PCs IN CD

Although the deep involvement of PCs has been clearly proved in the pathogenesis of IBD, cell‐specific treatments are poorly established.^95^ However, pathogenic pathways in PCs can still be targeted by many treatments regimens both specifically and non‐specifically.

To start with, PC phenotypes are potential CD indicator by determining prognosis and CD subtypes.^96^ PCs can be divided into five categories including normal, disordered, diminished, diffuse or excluded granule according to lysozyme‐positive secretory granule morphology.^96^ These phenotypes were proved having strong correlation between NOD2 and ATG16L1 T300A CD‐susceptible variants.^96^ Another reason to make PC phenotypes a CD indicator is the stability.^97^ PC phenotypes are consistent in involved and not involved areas. Moreover, PC phenotype is stable during the course of CD.^97^ Additionally, biopsy material can stably meet the criteria of PC phenotype analysis.^97^ All these advantages make PC a potential stable, practical and highly specific CD indicator.

Treatments based on NOD2 are well developed as NOD2 is considered to have the strongest correlation with CD.^98^ To begin with, defensin‐like drugs can be used to compensate AMP deficiency that is triggered by NOD2 defects without harming commensal bacteria.^99^, ^100^, ^101^ Additionally, faecal microbiota transplantation can be used to restore microbial composition imbalances and minimize NOD2’s effect on the microbiota.^98^, ^102^ Bacterial components such as MDP derivatives can be used to stabilize NOD2 stimulatory capacity.^103^, ^104^ Moreover, many other positive and negative regulators along with NOD2 signalling can be targeted to regulate the effect of NOD2.^98^ For example, RIP2 kinase inhibitors erlotinib and gefitinib can be used to block excessive inflammatory response.^105^

ATG16L1 may be another potential therapeutic target. For patients with the ATG16L1^T300A^ genotype, PPARγ signalling plays a role in regulating PC apoptosis and defects. Hence, PPARγ is a potential target for these patients.^106^ Furthermore, anti‐TNF‐α medication may be efficacious in CD patients harbouring the ATG16L1 SNP rs10210302. In an anti‐TNF‐α treatments study in which 570 IBD patients were retrieved and analysed, patients with ATG16L1 rs10210302 were specifically significantly more prone to use adalimumab (an anti‐TNF‐α medicine).^107^ As is mentioned above, one major reason is that the ATG16L1 polymorphism failed to prevent TNF‐α‐mediated PC necroptosis.

Wnt may also be a potential target for treatment. Normally, peripheral blood mononuclear cells (PBMCs) secret Wnt ligands which initiate Wnt pathway in PCs.^108^ As is mentioned above, Wnt pathway promotes PCs differentiation and regulates α‐defensins expression. However, in CD patients, PBMCs failed to secret enough Wnt ligands (Wnt1, Wnt3 and Wnt3a) to upregulate HD5/HD6 expression.^108^ Consequently, stimulating Wnt pathway may become an alternative CD treatment.

IFNL‐Stat1 signalling can also be a target for CD therapy. Glucocorticoids, such as dexamethasone and prednisolone, can not only attenuate inflammation in the intestine, but also suppress IFNL, which mediates PC death.^85^ Furthermore, tofacitinib is a stronger inhibitor of this pathway. The upstream of STAT1, JAK family, can be non‐selectively inhibited by tofacitinib. Accordingly, when one dose of tofacitinib a day was administered to Casp8^ΔIEC^ mice, PC death could be totally inhibited.^85^ Similarly, Filgotinib, another JAK inhibitor, which specifically inhibits JAK1, also strongly influences the IFN‐STAT1 pathway. Also, MLKL gene expression that directs PC death was found to be blocked with Filgotinib pretreatment in organoids.^85^

Targeting inflammatory pathways may be another way of treatment. As is mentioned above, IRGM prevents CD by inhibiting NLRP3 inflammasome. Hence, the administration of NLRP3 inhibitors on IRGM‐deficient IBD patients would be a useful approach to reduce the disease severity and symptoms.^49^ In CD, autophagy impairment results in degenerated mitochondria that drives intestinal stem cells (ISCs) transition into defect PCs. Reinforcing intestinal stem cell differentiation may improve intestinal epithelial function. According to a study, blocking glycolysis could antagonize PC dysfunction in CD.^109^ Dichloroacetate was added to the organoid culture medium to shift ATP generation from glycolysis to oxidative phosphorylation, which improved mitochondrial respiration and restored intestinal stem cell dysfunction.^109^ Furthermore, Wnt3a‐enriched medium could also reinforce intestinal stem cell differentiation and partially rescue organoid morphology.^110^ Interestingly, as a Wnt ligand, Wnt3a promotes defensins expression as well. Hence, further research on Wnt3a should be carried out.

In conclusion, potential treatments can be classified into four types. First, we can simply compensate Paneth cell functions by taking defensin‐like drugs or antibiotics.^111^ Second, environmental factors can be modified by smoking cessation, changing lifestyle and microbiota transplantation. Third, pathogenic pathways can be targeted by molecule‐targeted drugs to prevent Paneth cells defects or death. Finally, we can replenish Paneth cells by promoting ISC differentiation and intestinal transplantation.

## 11 DISCUSSION

The relationship between Paneth cells and Crohn's disease has been widely reported (Table 1). Recently, roles of Paneth cells in ileal Crohn's disease have emerged to discuss the risk factors and biologic behaviours. The lifestyle risk factors, AMPs, local microbiome influencing Paneth cells for ileal Crohn's disease are updated in this review.^112^ Thus, CD was believed to be a complex disease of PCs. Several links between CD and PCs are reported in a prior review.^113^ However, the connections and crosstalk for specific pathway and key nodes involved in the roles for Paneth cell acting on CD are still lacking to date. Thus, a clear list with signalling systems presented in this review is necessary. As mentioned in the prior review, certain PC antibiotic peptides play a role in preventing CD. One of the most prevalent PC defensins, HD‐5, has effective killing capacity against S. aureus as well as Gram‐negative bacteria, while the other, HD‐6, exhibited little antibacterial potential in vitro.^114^ Additionally, PCs also store several other innate antibiotic peptides (eg lysozyme, RegIIIγ and PLA2G2A) in cytoplasmatic granules.^115^, ^116^ Ileal CD is characterized by low Wnt Tcf‐4,^9^ which can induce PC differentiation. According to a prior study, a mild reduction in β‐catenin (a protein that a signalling cascades in the Wnt Pathway depend on) mRNA levels severely disrupted PC development.^117^ Also, CD is strongly related to NOD2 gene mutation (about a third of CD patients have the mutation^118^). NOD2 in CD was proposed to be linked to immunological dysregulation in monocytes^119^ and deficient PC antibacterial response (low defensin production).^113^ Finally, several other genes associated with CD were also reported. In detail, these include ATG16L1, which regulates PC autophagy, XBP1 which is expressed during ER stress, TLR9 (receptor of NOD2 in PCs) and KCNN4 (a potassium channel).^113^

**Table 1:** CD‐susceptible genes and their roles for Paneth cells

Susceptible genes	Functions in Paneth cells	Mediators	Reference
NOD2	Regulate α‐defensins; Participate in AMP sorting	NF‐κB, FGFR‐3, MAPK; LRRK2, RIP2, RAB2a	20, 21
ATG16L1	Promote exocytosis; Prevent necroptosis; Interact with environmental factors	LC3, ATG5, ATG12; TNF‐α,RIPK1; JNK, PPARγ	5, 34, 39, 46, 48
IRGM	Anti‐inflammation; Organize the core autophagy machinery	NLRP3, ASC, Caspase‐1, IL‐1β, SQSTM1/p62; ULK1, Beclin 1, AMPK, NOD2, ATG16L1	49, 51
Wnt	Regulate cell positioning, differentiation and maturation; Regulate α‐defensin expression	β‐catenin, Tcf/Lef family; TCF‐1, TCF‐4, LRP6	8, 9, 56
LRRK2	Mediate autophagy	Unknown	28
HDACs	Differentiation; Integrate commensal bacteria‐derived signals	Unknown	10, 11
Casp8	Prevent cell death	TNF‐α, TRADD, RIPK3, RIPK1, Notch, IFNL, STAT1	12, 81, 85
XBP1	ER expansion	IRE1, HAC1	91

After a decade of investigation, the profound mechanisms behind certain pathways are much better understood and new susceptible genes have been discovered. First of all, NOD2, a protein having been considered to have the strongest correlation with CD, was discovered to mediate various immune functions. In PCs, NOD2 controls α‐defensin expression through responding to bacteria stimuli, directing AMPs sorting, activating the NF‐κB Pathway and interacting with ATG16L1.^14^, ^22^, ^24^, ^120^ In other immune cells, NOD2 is necessary for autophagy in innate immunity.^98^ As a result, NOD2 is an important factor in clinical practice. Several targets and treatments based on NOD2 deficiency were discovered, which has already been mentioned. NOD2 is also a non‐negligible indication for a relevant treatment regimen and prognosis.^98^ NOD2 variants were identified as a risk factor for postoperative complications^121^ and the failure of antibiotic treatment in perianal fistulizing CD.^122^ Furthermore, NOD2 variants are also associated with loss of response to anti‐TNF treatments^123^ and biologic therapy of normal dose.^124^

Second, a highly specific autophagy gene, ATG16L1, was found to have a close relationship with PCs.^2^ ATG16L1 mediated autophagy is relevant to the degradation of several proteins in PCs.^5^ Thus, ATG16L1 variants may significantly disrupt PC homeostasis.^32^ The most important ATG16L1 polymorphism, ATG16L1^T300A^ (rs2241880, Thr300Ala), plays an important role in CD pathogenesis due to its defective function.^30^ Furthermore, ATG16L1^T300A^ accelerates its degradation by casp3 mediated processing,^125^ resulting in ATG16L1 deficiency. However, although the underlying mechanisms of ATG16L1 in PCs and CD are understood to a certain extent, treatments based upon it are still poorly established.

Additionally, other susceptible genes and pathogenic pathways have also been characterized to a greater extent. For example, in Wnt signalling, the function of TCF‐1’s in α‐defensin transcription was discovered subsequent to TCF‐4.^8^ IFN‐Stat1 signalling, a PC necrosis pathway, was proved to be related to Casp8 deficiency–induced CD.^85^ IRGM, another autophagy‐related CD‐susceptible gene, was found to inhibit the NLRP3 Inflammasome and exerted a protective effect on pyroptosis and gut inflammation in a mouse model.^49^

In the present review, we summarize new findings on the relationship between PCs and CD pathogenesis that are of great significance in scientific research and clinical practice. It is hoped that knowledge on susceptible genes, CD phenotypes and relevant treatment modalities can be integrated to improve the prognosis of CD patients.

## CONFLICT OF INTEREST

None.

## AUTHOR CONTRIBUTIONS

EY collected the paper and data, made conclusion analysis and drafted the manuscript; JS presented the idea of this paper, supported the funding, made conclusion analysis and drafted and revised the manuscript.

## Data Availability

Data sharing not applicable to this review as no datasets were generated or analysed during the current study.
